# A Novel Web App for Dietary Weight Management: Development, Implementation, and Usability Study

**DOI:** 10.2196/58363

**Published:** 2024-11-11

**Authors:** Ashleigh Oliveira, John Wolff, Nouf Alfouzan, Jin Yu, Asma Yahya, Kayla Lammy, Manabu T Nakamura

**Affiliations:** 1 Division of Nutritional Sciences University of Illinois at Urbana Champaign Urbana, IL United States; 2 Applied Research Institute Champaign, IL United States; 3 Department of Food Science and Human Nutrition University of Illinois at Urbana-Champaign Urbana, IL United States

**Keywords:** health application, weight loss, behavior change technique, BCT, online weight loss program, weight monitoring, meal planning, sustainable weight loss, dietary fiber, mHealth, mobile health

## Abstract

**Background:**

Online weight loss programs have ambiguous efficacy. There is a growing body of evidence that weight loss programs when combined with apps have better outcomes; however, many apps lack an evidence-based approach to dietary changes for weight loss and do not rely on a theoretical framework for behavior change.

**Objective:**

This study aimed to describe the development and the preliminary usability and acceptability testing of a web app that uses behavior change techniques (BCTs) to support users of a comprehensive online weight loss program.

**Methods:**

The weight loss program intervention components were nutrient and weight tracking charts that needed a remotely accessible and online format. The app was designed by nutrition researchers and developers in a collaborative effort. A review of BCTs in weight loss and web apps was performed as well as an assessment of user needs to inform the initial prototype. A preliminary app prototype, version 1.0, was provided to participants of a weight loss trial (N=30) to assess for feasibility of its use. A full app prototype, version 2.0, was feasibility and acceptability tested by trial participants (n=11) with formal feedback by Likert-scale survey and open-ended questions. In the final round of testing, a user group of scientists and developers (n=11) was selected to provide a structured 3-month review through which the group met weekly for collective feedback sessions.

**Results:**

The process resulted in a fully developed web app, MealPlot, by the Applied Research Institute, for meal planning and weight tracking that can be used by weight loss users and health professionals to track their patients. MealPlot includes a weight chart, a protein-fiber chart, and a chat feature. In addition, MealPlot has 2 distinct platforms, 1 for weight loss users and 1 for health professionals. Selected BCTs for incorporation into the app were goal setting, feedback, problem-solving, self-monitoring, and social support. Version 1.0 was used successfully to provide a functioning, online weight chart over the course of a 1-year trial. Version 2.0 provided a functional weight chart and meal planning page, but 8 out of 11 participants indicated MealPlot was difficult to use. Version 3.0 was developed based on feedback and strategies provided from user group testing.

**Conclusions:**

The web app, MealPlot, was developed to improve outcomes and functionality of an online weight loss program by providing a remote method of tracking weight, food intake, and connecting users to health professionals for consistent guidance that is not otherwise available in a traditional in-person health care setting. The final version 3.0 of the web app will be refined based on findings of a review study gathering feedback from health professionals and from actual weight loss users who are part of a clinical weight loss trial.

## Introduction

### Background

From 2000 to 2020, the prevalence of obesity rose from 30.5% to 41.9% in the United States [1]. Obesity in itself, as measured by BMI, may not be a health detriment, but if excess weight is intraabdominal adipose tissue, it is associated with conditions such as diabetes, hypertension, dyslipidemia, some types of cancer, coronary artery disease, and sleep apnea, all of which negatively affect the quality and longevity of life [2,3].

Methods of treatment for obesity include pharmacological, surgical, diet, or exercise [4]. Safety, sustainability, and weight loss magnitude vary among these treatment approaches. Pharmacological and surgical means of weight loss are highly effective in magnitude; however, these approaches have safety implications and are not accessible to everyone [5-7]. For example, only 0.5% of surgically qualified US adults every year will actually chose to undergo bariatric surgery [6]. In addition, studies following weight loss medication patients using glucagon-like peptide 1 have demonstrated significant weight regain once medication was discontinued [8]. Although a sustainable diet change is potentially a safer and more accessible treatment for obesity, maintaining a weight loss diet for a prolonged period followed by maintenance of this lost weight thereafter is challenging [9]. Many dietary weight loss methods rely on drastic alteration to normative eating that can be difficult to maintain resulting in insufficient magnitude of weight loss or weight regain [9]. Therefore, an individual must discover and build a sustainable, healthy eating habit for lifelong weight maintenance [10]. If excess weight is a result of ongoing diet and lifestyle habits, weight management programs that address such habits are an important component to losing and maintaining healthy weight [8,10-13].

Online weight loss programs are gaining popularity. More people have internet access and can enjoy the flexibility of an online platform that can be accessed anytime and anywhere that internet is available. However, despite popularity, online programs vary in efficacy and some research indicates they are less effective than in-person programs [14-16].

One component that may improve online weight loss program efficacy is incorporation of an app into the program [17-23]. In a meta-analysis of smartphone app efficacy for weight loss, 34 randomized controlled trials were included and then sub analyzed [24]. A total of 24 studies reported weight loss results. The pooled net estimated weight change at 3 months was –1.99 (95% CI –2.19 to –1.79) kg and at 6 months was –2.80 (95% CI –3.03 to –2.56) kg, respectively, as compared with controls [24]. Subgroup analysis (n=10) showed that the greatest weight loss at 6 months was seen when the intervention app was in combination with a behavioral intervention including health coaching as opposed to the app alone, –3.77 (95% CI –4.05 to –3.49) kg [24]. For those interventions including only the app (n=5), weight change was –1.59 (95% CI –2.37 to –1.83) kg [24].

While apps may contribute to improved outcomes, especially when combined with other intervention components, there remains significant heterogeneity in their effect [17]. According to literature review by Deniz-Garcia et al [25], reasons for discrepancies in app efficacy are due to variation in 4 significant categories including quality, usability, user engagement, and promotion of behavior change [25,26]. Quality, usability, and user engagement are specialty fields in web app development termed user interface/user experience. User interface/user experience web developers specialize in creating interfaces with user-centered design that are essential to making apps accessible within the intended population [26,27]. In regard to the promotion of behavior change, evidence indicates embedding multiple evidence-based behavior change techniques (BCTs) is an effective method [28,29].

BCTs are defined as the fundamental, observable, and reproducible elements within an intervention aimed at steering behavioral patterns [30]. Michie et al [31] configured the behavior change taxonomy of 93 BCTs to systematically and precisely identify methods of behavior change in interventions. The BCT hierarchy was constructed by a Delphi-type exercise whereby experts listed an extensive list of BCT labels from other published classification systems and then clustered them into groups. Classification of these techniques has allowed for methodical analysis of these interventions to pinpoint specific BCTs associated with intervention efficacy [28,30,32].

A systematic review of mobile health studies using BCTs from 2010 to 2021 was conducted to evaluate chosen BCTs on their efficacy in terms of adherence to intervention [28]. The review included 24 studies revealing associations between BCTs that were used in more and less effective studies that can provide some guidance for selection in mHealth development. BCT categories including feedback and monitoring and associations were used almost equally among effective and ineffective studies suggesting perhaps a null effect on adherence to the intervention [28]. The BCT grouping of feedback and monitoring includes feedback on behavior and self-monitoring of behavior and outcomes [28]. The BCT grouping of associations include prompts, cues, reward signaling, and methods of stimulus and associative learning. In addition, the study also found that BCT categories including goals and planning and personalization demonstrated a higher presence in effective than ineffective studies suggesting these may be significant BCT groupings to successful adherence. Goals and planning are defined as forms of goal setting and problem solving [28]. Personalization is not a traditional BCT, but an added BCT suggested by Dugas et al [33] in an extended taxonomy that is defined as the extent that a participant can tailor the intervention to their preferences.

Despite a potential null effect result of feedback and self-monitoring presented by Aguiar et al [28] review of adherence to mobile health interventions, these BCTs are associated with positive outcomes in weight-specific interventions. In a metanalysis and meta regression on BCTs preventing weight gain, those BCTs with a percentage effectiveness ratio of >50% included goal setting, self-monitoring, and feedback on behavior [34]. Some studies report mixed results to the feedback BCT for weight loss outcomes, which may be due to a lack of self-monitoring in these included studies [35]. In addition, weight loss patients with a higher frequency of monitoring their own weight, activity, and dietary intake show improved weight loss outcomes [36-38].

The BCT social support has ambiguously reported efficacy for online health interventions likely because they are less frequently cited as a tool in these remote apps and ambiguous as to their specific type of support [28,33]. Social support could be the social support of friends, family, community, or health care professionals [31]. Although there is limited evidence for social support in online interventions, the evidence for improved weight loss outcomes with incorporation of social support from a dietetic professional is apparent [39]. Patients with more frequent and longer intervention times with a dietitian have greater effect sizes in BMI change [39]. However, it is uncertain if encouraging contact to a dietitian by online health intervention as opposed to in-person may improve outcomes in these remote apps [40,41].

Apps that implement more than 1 BCT from the Michie et al [31] taxonomy appear to be more effective. In a systematic review of 18 mobile health interventions using BCTs to increase physical activity, those interventions using more than 1 BCT were more effective than those using one or less [30]. The most frequently used BCTs in the reviewed interventions were goal setting, problem solving, feedback, self-monitoring, social support, health information, and behavior practice or rehearsal [30]. While these are specific to increasing physical activity, similar BCTs are identified in weight management applications involving diet [29,42]. In a study of 35 commercial weight management apps available in China, higher-quality rated apps were associated with using more than 1 BCT of which goal setting, feedback, self-monitoring, and information or education were the most frequently cited. Quality in this study was measured using the Mobile Apps Rating Scale (MARS), a tool used to assess quality and likelihood of behavioral impact [42].

Despite having some guidance in BCT selection that may be effective for health interventions, strategically planning app development by embedding BCTs is a relatively new and rare concept that does not necessarily match the motivation for the development of most widely available commercial health apps [20]. Many commercial apps for weight loss are profit-driven and developed by companies to promote in-app purchases and sell user data to outside sources [43]. For example, MyFitnessPal, a food tracking app, embeds 1 BCT by allowing for self-monitoring of diet, but demonstrates no efficacy on its own for weight loss [44]. Jenny Craig and Nutrisystem have been suggested as more effective programs than others but rely on meal replacements that may not be financially affordable or sustainable [45]. Weight Watchers (presently WW International, Inc), which does not rely on meal replacements, but a point system, a simplified calorie counting instead, showed small but significant weight loss compared with control groups [45,46]. All 3 of these programs use apps for tracking, provide education, and incorporate some BCTs. Apps developed by researchers may be more likely to use BCTs, but due to limitations in funding or effective developer staff, these apps may be lacking in terms of quality and usability and are less likely to be available to the public. In addition, effective, evidence-based weight loss diets are not necessarily the trendy diets that commercial apps target to gain users [47]. In summary, although applying BCT to a weight management program is promising, currently there is no program that can be reliably used to treat obesity and its comorbidities.

Our long-term goal is to provide a dietary weight loss and maintenance program that is safe, sustainable, affordable, and accessible for a vast population. Nutrition researchers of the University of Illinois, Urbana-Champaign have been in the process of developing a program that includes online education sessions, nutrition coaching, weight tracking, and a uniquely flexible, satiating and nutrient-dense diet intervention focused on protein and fiber density. The program uses a BCT approach to dietary weight loss including goal setting, self-monitoring of weight and diet, self-efficacy in implementing sustainable dietary changes, and social support through communication with researchers and dietitians.

The original, in-person version of the weight loss program relied upon a hand-derived weight chart and protein-fiber (PF) chart as tools that were provided to individual participants on a weekly to monthly basis. The PF chart is a unique tool to visually demonstrate protein and fiber density of selected foods and meals on a 2D graph with a goal box for meal totals [48]. The PF chart is a meal planner that enables the user to create evidence-based safe and effective weight-loss and weight maintenance diets. For safe and effective weight loss, meal must contain higher protein and the same level of fiber while reducing calorie intake by a quarter to half of weight maintenance diet [49]. PF chart makes this difficult dietary change easier by visualizing protein to calorie and fiber to calorie values [48,50]. Instead of laborious daily calorie counting, the program uses a daily weight chart to monitor energy balance and set up weight goals. Food choices are flexible to users’ taste, budget, and cultural preferences by focusing only protein and fiber requirements. Preliminary research using the PF chart and weight chart in clinical trials is described in previous research by Lee et al [50,51]. To increase accessibility and potential outreach, it was decided to move the program to an online platform, EMPOWER, programmed by the Center for Innovation, Teaching Learning.

### Objectives

This study aims to develop a BCT-based web app, MealPlot, that will enhance behavior changes as an integral part of the online weight loss program, EMPOWER. The 3 pillars of the EMPOWER program are the nutrition education sessions, nutrition coaching by dietitians, and MealPlot app. MealPlot is intended to further increase implementation of behavior changes in multiple ways. An online PF chart as its primary component of MealPlot will enhance self-efficacy of patients in creating individualized meal plans. Other behaviors to be addressed in MealPlot includes daily weight chart (goal setting and self-monitoring) and messaging feature (increased support from registered dietitians). The preceding trials used manually derived charts including a weight chart displaying daily weights and a PF chart to display the protein and fiber density of meals in relation to a goal box. This paper reports the BCT approach used to develop the web app and the findings of formative interventions of this approach.

## Methods

### Overview

**Figure 1:**
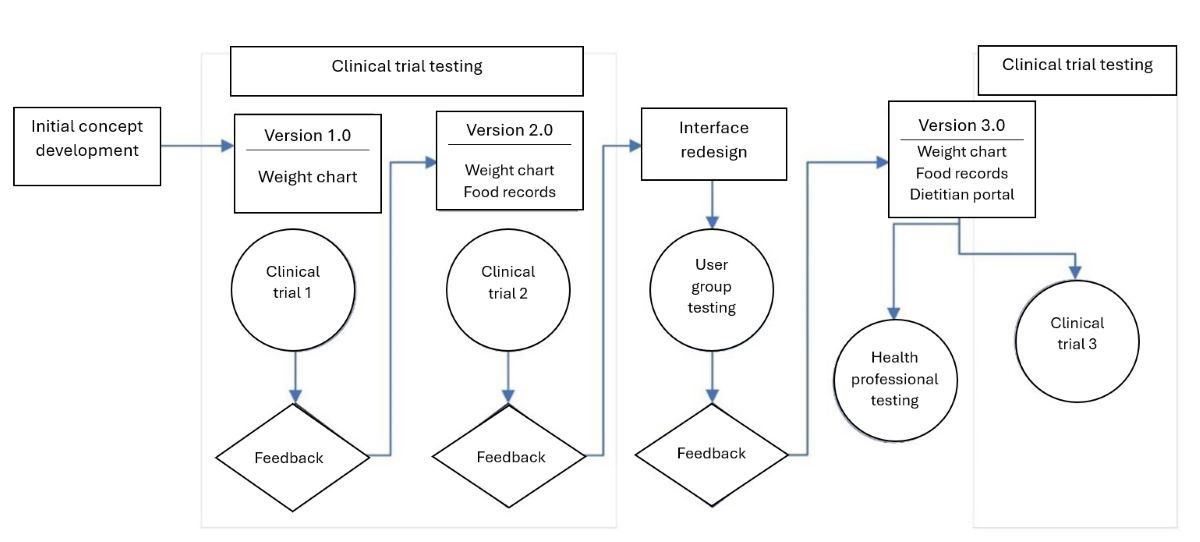
MealPlot intervention development model of the app testing and revision. The intervention development model of concept development through final version and methods of testing in-between version outputs. Version 1.0 was tested in a 2-year study (2021-2023) with participants (N=30) of a weight loss study using solely the weight chart. Version 2.0 was tested in a 1-year study (2022-2023) with participants (n=11) of a weight loss study using the weight chart and food record functions. User group testing was completed in 2023 (n=11). Version 3.0 testing is slated for 2024 among health professionals and weight loss trial participants.

**Figure 2:**
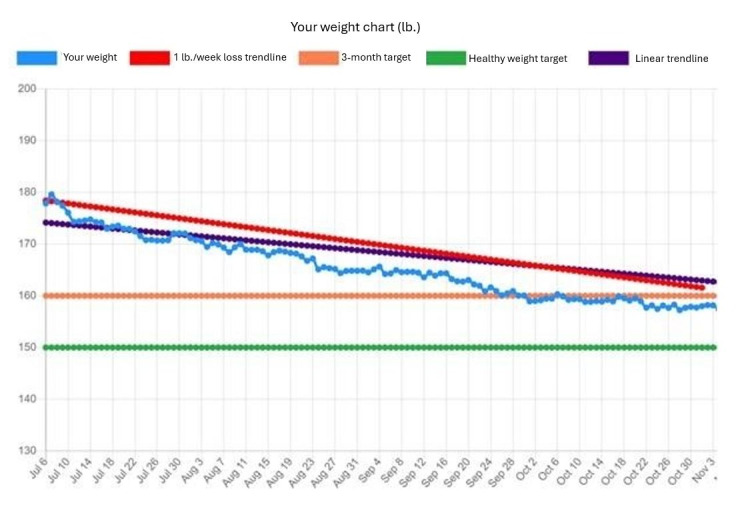
Weight chart example as portrayed in the MealPlot web app. Individual users connect their Wi-Fi–enabled scale to the web app to automatically load weights to the chart. The blue line represents the actual weights. The red line is a target trendline for the recommended 1 lb per week weight loss. The user’s goal weight is entered into the chart by their nutrition coach, represented by the orange line. Once a user is close to their healthy weight, a nutrition coach will add the health weight target, represented by a green line. Participants of MealPlot version 1.0 (N=30) and 2.0 (n=11) in a weight loss trial used this chart as a component of the app.

**Figure 3:**
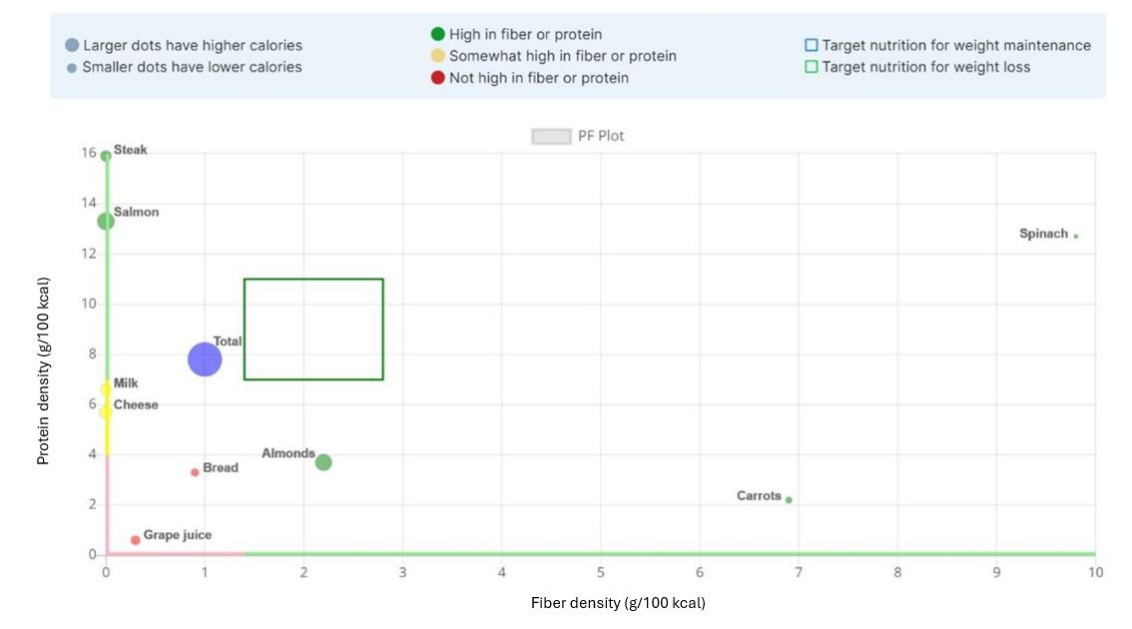
Protein-fiber chart as portrayed in MealPlot web app. Figure displays examples of foods plotted on the chart and the meal total. Individual foods are plotted with colors indicating their protein and fiber density. An individual food that is high in protein or fiber density is green, moderate in protein or fiber density is yellow, and low in protein or fiber density is red. The green box represents the target area for the total meal which is represented as a purple dot. Dot size indicates the number of calories consumed within the plotted meal. Meals with mostly green foods will have a total that falls into the target, while red foods will draw the total away from the target. Participants using MealPlot version 2.0 in a weight loss trial (n=11) used this chart as a component of the app. PF: protein-fiber.

The stages of MealPlot development and testing are described in the MealPlot intervention development model (Figure 1). MealPlot was conceptually designed, piloted in 1-year weight loss clinical trials, and user group tested throughout which feedback in between each round of testing was used to improve upon the previous version. Version 1.0 included a fully developed weight chart and weight tracking feature, which was tested in a clinical trial for feasibility (Figure 2). Version 2.0 involved all planned web app features, including the weight chart, meal planning, and 24-hour record submission features with an interactive PF chart (Figure 3). The fully developed web app (version 2.0) was tested by 2 methods. First, 11 participants enrolled in an online feasibility health intervention using our MealPlot web app provided feedback by an acceptability interview and survey. Second, a structured user group testing was conducted. Feedback from both the feasibility and acceptability study and the user group testing resulted in creating a new interface and substantive updates to the search database. In the final future stages, version 3.0 will undergo user testing in a clinical trial and a review by nutrition professionals.

### Ethical Considerations

This study includes a secondary analysis from clinical trial participants who provided feedback of MealPlot versions 1.0 (IRB #18069) and 2.0 (IRB #22642). The data used in this analysis were anonymized and deidentified according to the Health Insurance Portability and Accountability Act (HIPAA). Ethics approval was obtained from the University of Illinois Institutional Review Board for the primary studies and the use of these studies in this secondary analysis. No compensation was provided for participation. All participants provided informed consent before participation. Data within the MealPlot web app was protected by data encryption techniques. Personal data were available within the app to the participants of the study.

### Preparatory Phase

In the preparatory phase, a comprehensive literature review was conducted, including controlled trials and review papers on BCTs and apps applied to health interventions. The BCTs chosen to incorporate into the MealPlot app were considered a customary practice of health interventions and associated with successful outcomes. These BCTs included those most associated to effective online interventions by Aguiar et al [28] including goal setting and problem solving. Although Aguiar et al [28], also indicated feedback and monitoring as potentially null, these feedback and self-monitoring were included based on significance found of these BCTs in interventions that are specific to weight [35-38]. In addition, the social support, BCT was included even though there are ambivalent reports of its efficacy [28]. We included this BCT due to the significance of social support from dietetic counsel for weight loss and maintenance success [39]. Finally, the BCT personalization was decidedly not included in the development planning despite its positive effect on intervention adherence due to programming limitations with the first round of development; however, it was considered an addition for potential future improvements [28].

The components or pages selected for initial development were identified through multiple focus groups composed of nutrition researchers and developers. Developers were hired from the Department of Applied Research Institute at the University of Illinois. Nutrition researchers had experience running the weight loss program in its in-person version and were able to identify those parts of the intervention that would be enhanced by an online web app.

An open source, free web app instead of mobile app was chosen to provide the most inclusive platform possible (mealplot website) that can be accessed internationally. Web apps can be flexibly used in any web browser and on any device capable of internet connection, while mobile apps require iOS (Apple Inc) or Android (Google) platforms [52].

### Development Phase

**Figure 4:**
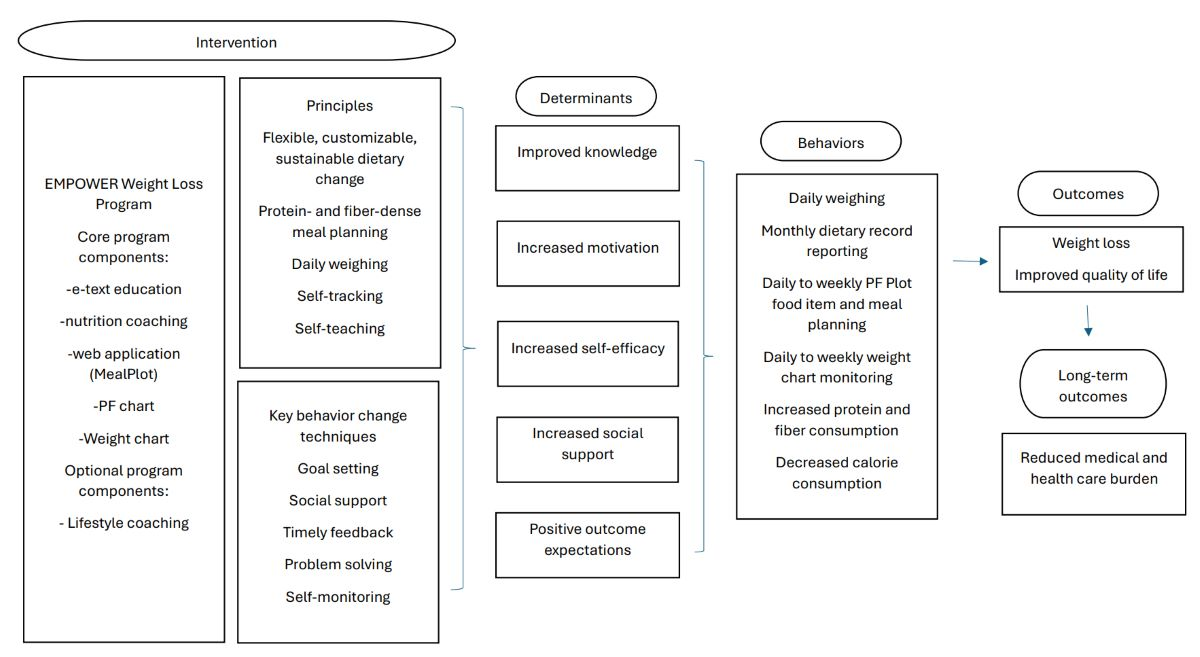
MealPlot and EMPOWER conceptual framework portraying the intervention components, determinants, behaviors, outcomes, and long-term outcomes as conceptualized by nutrition researchers of the EMPOWER weight loss program and app programmers. The framework was used to inform the development of the app and to create guidelines for future app and program assessments. PF: protein-fiber.

In the development phase, a web app prototype was created (Figure 4). The prototype included a version for users and for researchers or nutrition coaches. The user version included meal planning and 24-hour record tools, weight chart, and profile page where users could grant access to researchers to view their data (Table 1). The researcher version had an additional researcher page where data could be downloaded from users who had granted them access.

**Table 1:** MealPlot prototype pages and features displaying the planned pages and subcomponents to each page of the app with included descriptions. MealPlot version 1.0 included the weight tracking, data downloads, and profile. MealPlot version 2.0 included all pages and components listed.

Page and component			Content description
**Meal planning**			
	Food search toolbar	The food search toolbar has a text bar for unique text entry.	
	Food search categories	A toolbar list of food search categories includes common food groupings and drop-down menus of subcategories for selection as an alternative to searching.	
	Food database	Food items and nutrient information are populated from data extracted by USDA^a^ Food Central database.	
	PF^b^ chart	The PF chart is below the search feature. The chart automatically populates added foods indicating their protein and fiber densities by x- and y-axis and color coding.	
	One day record	The 1-day record queries intake of food item, intake amount, time, place, and date of consumption and records a log as well as displays on the PF chart.	
**Weight tracking**			
	Wi-Fi scale connection portal	The weight chart page has a button connecting the Wi-Fi scale account to MealPlot allowing for automatic pulling of scale data to MealPlot.	
	Manual weight entry	For users without Wi-Fi scales, weights can be entered manually with the weight and date.	
	Weight chart	The weight chart automatically populates the users’ actual weights in blue on a graph with a 1 lb/week weight loss trendline. Individualized target weights can also be added to the graph.	
**Data downloads**			
	Weight and one day record data	Users with the researcher or coach designation that are granted permission by users to view data have buttons to download weight and nutrition data of the user in a requested period to a Microsoft Excel output.	
**Profile**			
	User email and password	The profile page has the user’s account information including name and email. At this page, users can update their information and change their password.	
	Data permissions	A checkbox for granting access to weight and nutrition data to researchers is located below account information.	

^a^USDA: United States Department of Agriculture.

^b^PF: protein-fiber.

The selected BCTs (goal setting, social support, feedback, problem solving, and self-monitoring) were incorporated into the app design. The goal setting was designed as specific, measurable, achievable, relevant, and time-bound (SMART) where possible [53]. SMART goals were incorporated into the meal designing tools through an online PF chart and color-coding of individual foods. The specific and measurable goals included protein and fiber densities for individual foods and meals. A stoplight themed color-coding system was used to label foods as either high, medium, or low protein and fiber density. The PF chart was also included with a target box (Figure 3). Users self-monitor their dietary habits by searching and inputting their consumed or planned foods, resulting in immediate feedback on the PF chart to see if selections were within a target box. Weight tracking and weight goals were programmed into an online weight chart (Figure 2). The weight chart was designed to automatically collect users’ weights by Wi-Fi–enabled scale. Realistic 3-month weight goals could be set by researchers and entered into the chart.

The social support BCT was established by making the app a portal between nutrition coaches and weight loss participants. Weights and food records of weight loss participants could be viewed and analyzed by their nutrition coaches, thereby facilitating meaningful discussions during coaching sessions.

### Feasibility Testing of Version 1.0 of MealPlot

#### Purpose

The first fully online cohort of the weight loss program were early adapters of the web app. The purpose of introducing the app to users in this stage was to begin feasibility testing of the weight chart feature and overall functionality of the site. This first version of MealPlot included a fully functioning weight chart and basic profile utilities. Other components of the web app as drafted in the prototype, the PF chart, meal planning, and 24-hour record submission were in development.

#### Methods

Study participants who had consented at baseline and remained in the weight loss program for feedback of MealPlot version 1.0 were eligible. Participants were recruited through word of mouth, newsletters, and posters in an Illinois health clinic in collaboration with cardiac rehab physicians. REDCap (Research Electronic Data Capture; Vanderbilt University) software was used to collect eligibility criteria and consent. Exclusion criteria included current pregnancy or lactation, severe chronic disease diagnoses, and previous or planned bariatric surgery. Participant characteristics included being of age 18 to 75 years old and a BMI >25 kg/m^2^. Recruitment for trial participants of MealPlot version 1.0 was in July 2021 and lasted 4 weeks.

Version 1.0 was tested in a 1-year clinical trial for feasibility. Participants of the trial (N=30) took home a Wi-Fi scale, created a MealPlot account, and connected the Wi-Fi scale to the MealPlot platform through a connective portal within the app.

#### Results

Beyond minor technical difficulties, the features of the weight chart and data downloads of the participants’ weights by researchers were fully functional and used as a major component of the clinical trial. The weight chart automatically populated the users’ weights drawing data from a Wi-Fi scale or weight could be entered manually. A data permissions feature allowed users to grant researchers access to their weight data from which researchers could download the users’ weights and update their weight charts to include individualized and adaptable goals. While some participants needed technical assistance, all participants could complete this process. Participants then granted researchers access to view their weight data through which researchers were able to give participants individualized goals on their weight charts and download their daily weights for feedback and analysis. The 2-year study was completed in June 2023.

### Feasibility and Acceptability Testing of Version 2.0 of MealPlot

#### Purpose

The second version of MealPlot contained all prototype components of the web app including the weight chart, meal planner, and 24-hour record submission. The PF chart was embedded within the meal planning and 24-hour record features, displaying the chart for the user to see as they entered meals. With a fully complete web app, the purpose of feasibility and acceptability testing in a small 1-year clinical weight loss trial was to gather feedback from an underserved population that was experiencing disproportionately higher rates of obesity.

#### Methods

Study participants who had consented at baseline and remained in the program for feedback of MealPlot version 2.0 were eligible with characteristics of age 18 to 75 years old and a BMI >25 kg/m^2^. Exclusion criteria included current pregnancy or lactation, severe chronic disease diagnoses, and previous or planned bariatric surgery. Clinical trial participants were recruited through posters, flyers, and social media in Illinois, United States with the aid of 3 extension regional offices. REDCap software was used to collect eligibility criteria and consent. Recruitment for trial participants of MealPlot version 2.0 occurred in May 2022 for 3 weeks.

Version 2.0 was tested in a feasibility and acceptability study of the EMPOWER weight loss program in rural and underserved areas (n=11). The 1-year trial included an acceptability survey and interview (Multimedia Appendix 1). The acceptability survey and interview included 4-5 questions per intervention component (overall intervention, MealPlot, education sessions, nutrition coaching, and lifestyle coaching). Survey questions inquired on the level of satisfaction, helpfulness, and ease of use as a Likert scale and were provided to participants by REDCap. The final question was an open-ended interview question inquiring on the opinions of each intervention component and was presented as a one-on-one interview by telephone with the lead researcher. Interviews were recorded and transcribed verbatim. Acceptability survey question responses were averaged for each question. The interview transcriptions were analyzed thematically using 6 steps of transcription and familiarization, identifying keywords, creating codes, developing themes, conceptualizing the themes, and then road mapping the results [54].

#### Results

Despite limited internet access at participant homes, web app use was not impeded; however, the acceptability survey indicated MealPlot to be the least satisfactory or helpful component of the overall EMPOWER program. On average, participants indicated their level of satisfaction to be 2.82 (SD 0.83) with the MealPlot web app with 1 being unsatisfied and 5 being extremely satisfied. The level of helpfulness was on average 2.55 (SD 1.08) with 1 being not helpful and 5 being extremely helpful. In addition, 73% (8/11) of participants indicated that MealPlot was difficult to use. Interviews revealed that while the daily weighing and use of the weight chart was acceptable, the meal planning tool needed improvement. Areas for improvement included the performance of the search feature of the food database, the availability of foods in the database, and the comprehensibility of the interface were major themes of the interview. Thematic analysis and substantive quotes from the interviews are shown in Textbox 1.

**Illustrative quotes and themes from a 3-month acceptability survey of clinical trial participants partaking in a 1-year weight loss study (n=11) that used MealPlot version 2.0 regarding improvements to the program components (“How can we improve our program to make your experience better?”).**

**Search feature and database**

- “Plotting food is difficult because of search function, the database brings up unusual foods instead of simplest food.”
- “The food search feature is difficult to use…it doesn’t give an option for just a lot of common foods.”
- “Plotting food is difficult because of search function, the database brings up unusual foods instead of simplest food.”

**User interface**

- “...can be tricky.”
- “It’s confusing and cumbersome to navigate.”
- “…its challenging and doesn’t make sense.”

### User Group Testing

#### Purpose

Before providing the MealPlot web app to another cohort of weight loss users, it was determined that the user interface and search features of the food database needed improvement and quality testing. Methodical user group testing on a variety of platforms by a diverse group of testers may ensure a higher success of adoption and acceptability in the general population [55].

#### Methods

A diverse team of user testers and web app developers were recruited through email and word of mouth. Testers included university students studying food science and human nutrition (n=6), nutrition researchers (n=3), and web app developers (n=2).

Participants collaborated in a structured user group testing over the course of 3 months. User group testers were required to learn about the comprehensive EMPOWER weight loss program to use the web app from both the perspectives of a weight loss user and a researcher or nutrition coach. Group meetings occurred weekly, deliberating on the findings of the assigned testing area. User group testers also completed a weekly Qualtrics (Silver Lake) survey regarding questions of the assigned testing area to ensure adequate participation was completed.

#### Results

A comprehensive review of feedback from both the feasibility and acceptability study and the user group testing resulted in an expanded food database, improved search capacity, and new interface.

The original database was taken from United States Department of Agriculture (USDA) Food Central [56]. While comprehensive, accurate, and reliable as compared with other food databases, limitations were discovered. Some of these limitations included uncommon terminology used for foods or a lack of popular cultural terms.

To compensate for missing items and variation in terminology, the database was augmented with foods from 31 common restaurants in the Midwest, including 9038 individual items. Nutrient information was collected from the restaurant websites. In addition, 1443 foods that were present in the originally derived database of the USDA were dually entered with popular culture terms.

The functionality of the database was improved through increasing search term flexibility. Users could search for food items containing multiple words in any order, use plural or singular terms, and still retrieve a result. Finally, the accuracy of the retrieved result was improved by ordering the retrieved results from the shortest items, matching all searched words to the longest.

**Figure 5:**
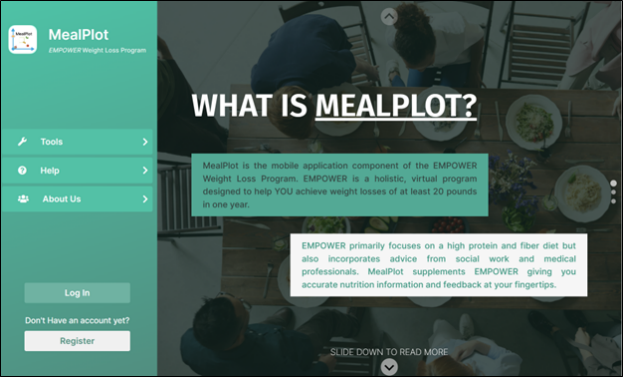
Screen capture of the MealPlot version 3.0 home page.

**Figure 6:**
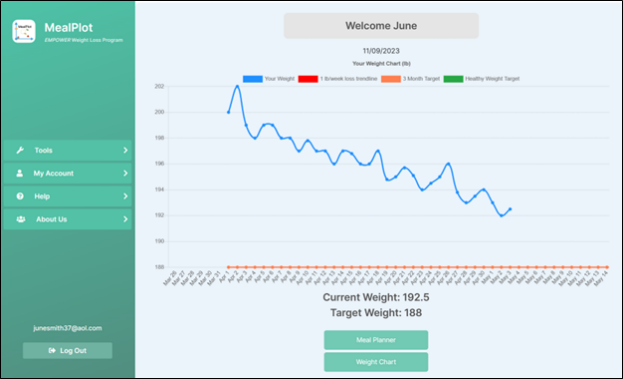
Screen capture of the MealPlot version 3.0 dashboard.

**Figure 7:**
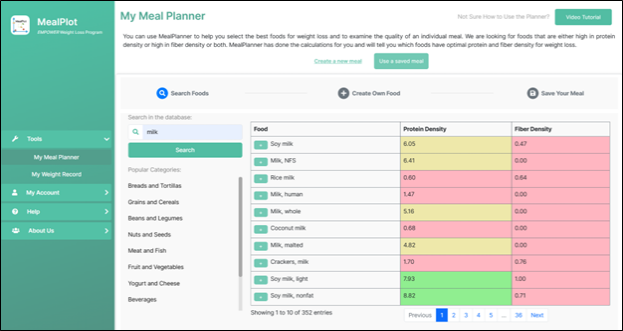
Screen capture of MealPlot version 3.0 meal planning and one day record page.

**Figure 8:**
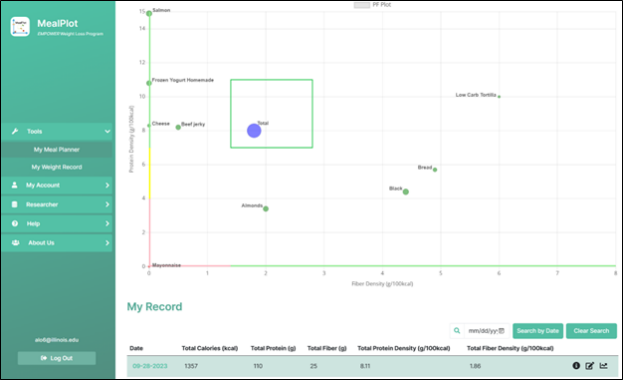
Screen capture of MealPlot version 3.0 protein and fiber chart.

The MealPlot 3.0 interface was redesigned by the 2 web app developers who had also participated in the user group testing. Updates included (1) a dashboard succinctly summarizing significant information, (2) an integrated meal planning and one day record, (3) a chat feature between researcher and user, and (4) an enhanced researcher portal displaying client PF charts and weight charts in addition to the already present capacity to download data to Microsoft Excel (Table 2). Screen captures of MealPlot 3.0 home page, dashboard, meal planning and one day record, and protein and fiber chart are displayed in Figures 5-8.

**Table 2:** MealPlot version 3.0 pages and feature updates displaying the planned pages and subcomponents added to the additional app with included descriptions for MealPlot version 3.0.

Page and component			Content description
**Dashboard**			
	Landing page	Immediately upon login, the dashboard appears with the user’s name, date, current weight, weight goal, weight chart, and quick access buttons to the meal planner and weight chart pages.	
**Meal planning**			
	Integrated meal planning and one day record	The meal planning and logging of foods were integrated. As a user inputs foods for meal planning, they have the option to log them to their planner as a record instead of starting over on a separate one-day record page.	
**Chat**			
	In-app messaging	A message feature is accessible by the left navigation toolbar. When messages are present, a red notification appears. Messages are allowed between researcher or coach and participants with granted access in an SMS text message format within the app.	
**Nutrition coach portal**			
	Weight and one day record data	Users with researcher or coach designation can complete all tasks unique to this role within the researcher portal.	
	PF^a^ chart, weight chart, chat	In addition to data downloading, researchers can view the PF charts and weight charts of their accessible users and initiate chats.	

^a^PF: protein-fiber.

## Discussion

### Principal Findings

The MealPlot web app is a complementary tool to a comprehensive online weight loss program EMPOWER. MealPlot uses BCTs with the intention of supporting patient-led dietary changes through color-coding of foods, SMART goals of nutrients and weight, self-monitoring of diet and weight, instant visual feedback, and social support from an actual health professional.

Many weight loss apps act as stand-alone approaches to weight loss focusing on logging foods to plan a calorie deficit. MealPlot is not intended for use as a sole component to the weight loss journey, but instead as an additive component to education and professional support, which has been shown to be a significant factor for weight loss in our background research [10,16]. In addition, while a calorie deficit is necessary for weight loss, MealPlot guides users towards nutrient-dense foods that naturally lead to a reduction in calories. Instead of continually relying on daily tracking, users are intended to discover new foods that meet their nutrient objectives and wean from using the app over time.

The final development of the app is a result of several rounds of preliminary testing and revisions, accounting for user perceptions and professional expertise of functionality and helpfulness. We have developed all the materials required for a fully comprehensive online weight loss program including the MealPlot web app, online nutrition education sessions, and a manual for health professionals on how to use the program with their patients. Health professionals with patients intending to lose weight have the full capacity of using the web app to monitor their patients remotely using MealPlot.

### Future Directions

Version 3.0 of MealPlot has the capacity for adoption into a health care setting using nutrition professionals as administrators and weight loss users as patients or clients. This new platform design has 2 portals, one for users and the second for nutrition professionals or nutrition researchers. The user portal is for individuals who are intending to manage their weight. The other portal intends for nutrition professionals to monitor the weight loss users on the app. The purpose of this intervention is to enhance the weight loss outcomes of users of the online EMPOWER program.

The final intervention uses MealPlot as a component of a comprehensive online weight loss program. EMPOWER incorporates online education sessions, coaching by nutrition health professionals, and the use of the MealPlot web app for meal planning and weight tracking. Users desiring to lose weight begin by taking the first few sessions of online nutrition education introducing concepts of the intervention. After learning about the significance of protein, fiber, and daily weighing, users are instructed to begin making dietary changes and self-monitoring their weight with the aid of the MealPlot web app. Tutorials on setting up the Wi-Fi scale, planning meals, and chatting with health coaches through the app are provided with short videos. Weight loss users are encouraged by the online homework and their health coaches to experiment on the app so that they can discover protein and fiber-dense meal selections that will fall into their designated goal target boxes of the PF chart. Color coding of foods and meal output targets provides immediate feedback on the chart. In addition, individual goals are prescribed on the weight chart where users can see their daily weights in comparison with these goals.

Nutrition coaches on the platform request access to their patients’ data which is prompted to the weight loss user. If the user allows the coach access, the coach and weight loss user can chat, and the coach can view the users’ data. Throughout the intervention, the coach will view the patient’s weight loss progress and food selections providing feedback and encouragement in the chat. In addition, the coach can request for 24-hour records that the patient can input through the app for immediate download and feedback. The overall intervention is meant to flexibly provide 3 months of nutrition education sessions followed by use of the app for as long as the weight loss user needs to achieve and maintain a healthy weight.

### Limitations

Within this study, we aimed to comprehensively develop and test the MealPlot web app; however, we acknowledge some limitations. The app has not been tested in its latest version by weight loss users. In addition, the app testing has not assessed its value as compared with other weight loss apps that are already available. To address these limitations, the next step will include research that collects perceptions of the app from weight management professionals and weight loss patients through a qualitative study on dietitian perceptions and surveys from EMPOWER weight loss participants. Interviews will collect qualitative perceptions. Surveys such as the MARS will be used to benchmark the app’s quality and likelihood of behavioral impact against other health apps. Cost analysis is also required to govern the sustainability of the MealPlot app.

### Conclusions

This study demonstrates how an evidence-based approach can be applied to the creation of an app for health interventions to support a comprehensive weight loss program. The MealPlot web app was developed to implement specifically selected BCTs to an evidence-based dietary weight management program. Future refinements will be guided by evidence of findings from interviews by health professionals and usability surveys of the app by both weight loss users and health professionals. The efficacy of the MealPlot app will be tested by a clinical trial of the EMPOWER weight loss program in the future.

## Appendix

Multimedia Appendix 1 Acceptability Survey and Interview.

## Abbreviations

BCT: behavior change technique
HIPAA: Health Insurance Portability and Accountability Act
MARS: Mobile Apps Rating Scale
PF: protein-fiber
REDCap: Research Electronic Data Capture
SMART: specific, measurable, achievable, relevant, and time-bound
USDA: United States Department of Agriculture

## Data Availability

The datasets generated during and analyzed during this study are available from the corresponding author on reasonable request.
